# Properties of Cast Films Made from Different Ratios of Whey Protein Isolate, Hydrolysed Whey Protein Isolate and Glycerol

**DOI:** 10.3390/ma6083254

**Published:** 2013-08-02

**Authors:** Markus Schmid

**Affiliations:** 1 Fraunhofer-Institute for Process Engineering and Packaging IVV, Giggenhauser Strasse 35, Freising 85354, Germany ; E-Mail: markus.schmid@ivv.fraunhofer.de; Tel.: +49-8161-491-526; Fax: +49-8161-491-555; 2 Chair of Food Packaging Technology, Technische Universität München, Weihenstephaner Steig 22, Freising 85354, Germany

**Keywords:** whey protein isolate, hydrolysed whey protein isolate, barrier properties, mechanical properties, surface tension, yellowish coloration

## Abstract

Whey protein isolate (WPI)-based cast films are very brittle, due to several chain interactions caused by a large amount of different functional groups. In order to overcome film brittleness, plasticizers, like glycerol, are commonly used. As a result of adding plasticizers, the free volume between the polymer chains increases, leading to higher permeability values. The objective of this study was to investigate the effect of partially substituting glycerol by hydrolysed whey protein isolate (h-WPI) in WPI-based cast films on their mechanical, optical and barrier properties. As recently published by the author, it is proven that increasing the h-WPI content in WPI-based films at constant glycerol concentrations significantly increases film flexibility, while maintaining the barrier properties. The present study considered these facts in order to increase the barrier performance, while maintaining film flexibility. Therefore glycerol was partially replaced by h-WPI in WPI-based cast films. The results clearly indicate that partially replacing glycerol by h-WPI reduces the oxygen permeability and the water vapor transmission rate, while the mechanical properties did not change significantly. Thus, film flexibility was maintained, even though the plasticizer concentration was decreased.

## 1. Introduction

Protection against negative impact from the environment is the primary function of food packaging [1]. The barrier requirements of food products are strongly dependent on the composition and ingredients of the product. Especially for products containing high amounts of unsaturated fatty acids or oxygen sensitive vitamins, the protection against oxygen is one determining factor that guarantees the maximum shelf life of the products [2]. For that reason, modified atmosphere packaging (MAP) concepts are commonly used for sensitive foods. MAP often utilizes nitrogen gas as an inert replacement for air, where oxidation is undesirable. The required oxygen barrier properties in order to maintain the low oxygen concentration in the headspace of MAP packages are often provided by petroleum-based materials, like PA (polyamide), or engineered oxygen barrier polymers, such as ethylene-vinyl-alcohol (EVOH) or polyvinyl alcohol (PVOH), in multilayer structures [3]. These passive barrier functions can be additionally combined with active barrier layers in order to support the passive barrier and to reduce harmful oxygen in the headspace of a package [4].

To focus more on sustainability, there is an increase of research activities on renewable materials. However, all these activities only contribute to sustainability, if it can be ensured that the protection of the packed goods is assured, as the value of the resource input into and tied up in foodstuffs is far higher than that of those in packaging [5]. Thus, it is strongly desired that novel bio-based materials provide similar barrier and mechanical properties in order to be suitable to replace petroleum-based materials. A possible alternative to achieve suitable mechanical, as well as high oxygen and water vapor barrier properties, is coating of polymer films with whey protein. Whey protein is a by-product of cheese manufacturing and able to act as an excellent oxygen barrier material that also provides suitable mechanical and water vapor barrier properties [6,7,8,9,10,11,12,13].

Unplasticized whey protein isolate (WPI)-based films are brittle, due to several chain interactions caused by a large amount of different functional groups and, therefore, not applicable for industrial application. These chain interactions consist of disulfide bonding, hydrogen bonding, hydrophobic interactions and electrostatic forces between protein chains and, typically, lead to brittle films [14]. In order to overcome film brittleness, external plasticizers, like glycerol, are commonly used. They reduce intermolecular protein chain-to-chain interactions, increase polymer chain movability and, thus, increase film flexibility [15]. Nevertheless, these interactions are the reason for its desired oxygen and water vapor barrier performance. Thus, as a result of adding plasticizers, the permeability values increase, due to the increased chain movability and a higher free volume in the protein network [16,17,18]. However, increased oxygen permeability and water vapor transmission rates are not desired for most sensitive food products, which have to be protected against oxygen and/or water vapor [16,19,20,21,22]. The incorporation of plasticizers additionally decreases the elastic modulus (EM) and tensile strength (TS), leading to more flexible films, while the film elongation (E) is increased. Another appropriate approach to improve film flexibility is to reduce the molecular weight (M_W_) of the polymer. This effect can be explained by reduced intermolecular forces along the polymer chains, increased polymer chain end groups and increased free volume in the polymer matrix [23]. In the case of whey proteins, Sothornvit and Krochta [17,18] compared the techno-functional properties of films made from denatured WPI and hydrolysed whey protein isolate (h-WPI) having a degree of hydrolysis (DH) of 5.5% and 10%, whereby DH is defined as the percentage of peptide bonds cleaved [24]. They concluded that h-WPI makes good films with oxygen permeability and water vapor transmission rate values similar, but with more flexibility, than WPI films at the same glycerol (Gly) content.

Schmid *et al.* [25] followed and quite recently published in this journal a similar approach. They studied, in contrast to Sothornvit and Krochta [17,18], films made from mixtures of WPI and h-WPI having a DH of 10%. Schmid *et al.* [25] showed that increasing the h-WPI content, thus decreasing the average protein, M_W_, in WPI-based films at constant Gly concentrations, also significantly increases film flexibility, while maintaining the barrier properties, compared to films entirely produced from h-WPI. The present study is a consistent continuation considering these facts in order to create films having increased barrier performance while maintaining film flexibility.

Thus, the objective of this study was to investigate the effect of partially substituting Gly by h-WPI in WPI-based cast films on their mechanical, optical and barrier properties. Therefore, Gly and WPI were partially replaced by h-WPI in WPI-based cast films. The novelty of this approach is given by the fact that films made from mixtures with different ratios of WPI, h-WPI and glycerol were analyzed in contrast to former studies [17,18], where either non-hydrolysed WPI, fully or partially hydrolysed films were analyzed at different glycerol ratios.

## 2. Materials and Methods

### 2.1. Materials

BiPro WPI with a minimum protein content on a dry basis (total Kjeldahl nitrogen conversion factor, *N* × 6.38) of 95% and BioZate 3 h-WPI with a degree of hydrolysis (DH) of 10% and a minimum protein content on a dry basis (*N* × 6.38) of 94% by Davisco Foods International (Le Sueur, Minnesota, MN, USA) were used to produce the whey protein isolate-based films. The M_W_ distribution of the h-WPI was as follows (given as percent of peptides):

- 60%–75%: <2 kDa
- 15%–25%: 2–5 kDa
- 5%–15%: 5–10 kDa
- 1%–10%: >10 kDa

Data for degree of hydrolysis, M_W_ distribution and protein content were provided by Davisco Food International. Gly was used as the plasticizer and was purchased from Merck Schuchardt OHG (Hohenbrunn, Germany).

### 2.2. Film Casting

Aqueous solutions (pH 7) of distilled water, WPI or h-WPI were prepared and stirred for 30 min at 23 °C in the electric stirrer Thermomix 31-1 (Vorwerk Elektrowerk GmbH & Co. KG, Wuppertal, Germany). The flask volume of the electric stirrer was 1.5 L, and the volume produced per sample batch was 1 L. Gly was added, and the resulting aqueous solutions were stirred for another 30 min with the magnetic stirrer, MR3001 (Heidolph Instruments GmbH & Co. KG, Schwabach, Germany), at 200 rpm. Finally, the WPI solutions were treated for 15 min in an ultra-sonic bath at a frequency of 37 kHz and 23 °C to enable removal of foam and bubbles using a pipette after the ultra-sonic treatment.

In order to analyze the effect of partially substituting Gly by h-WPI in WPI-based cast films on their film properties, 1% (w/w) WPI and 1% (w/w) Gly was substituted by 2% (w/w) h-WPI, leading to the following set of formulations (Table 1).

**Table 1:** Ratios of whey protein isolate (WPI), hydrolysed whey protein isolate (h-WPI) and glycerol (Gly) in aqueous solutions/formulations.

Formulation number	WPI [%] related to total (w/w)	Gly [%] related to total (w/w)	h-WPI [%] related to total (w/w)	Total protein [%] related to total (w/w)	Gly [%] related to total protein (w/w)
1	20	13.34	0	20	66.70
2	19	12.34	2	21	58.76
3	18	11.34	4	22	51.55
4	17	10.34	6	23	44.96
5	16	9.34	8	24	38.92
6	15	8.34	10	25	33.36

The ratios of Gly, WPI and h-WPI are also displayed by the *x*-axis labelling of the figures. The dry matter content of the formulations was at a constant 33.34% (w/w) and the water content at 66.66% (w/w), respectively. 

Since the formulations were not heat treated previous to the casting process, it was possible to achieve these high dry matter contents without gel formation, which usually occurs on heat denatured whey protein formulations, due to induced protein unfolding and exposure of internal sulfhydryl groups, which promote intermolecular disulfide bond formation [26]. According to Schmid *et al.* [27], it is possible to use native whey protein during the coating/casting process step, whereas the necessary denaturation of the protein occurs during the drying step. Thus, native whey protein formulations can be applied with higher solid contents compared to fully denatured whey protein formulations, leading to solutions that can easily be used for film casting and coating processes in all classical processes, leading to a considerable savings of process energy.

For film casting in the present study, 9.5 mL of the above mentioned native formulations were filled into petri dishes (120 mm × 120 mm × 14.5 mm) made of polystyrene (PS) in order to form films with a dry film thickness of approximately 200 µm. This roughly corresponds to a dry casting weight of 200 g/m^2^. To achieve a homogenous distribution of film thicknesses, eight-shaped movements with the petri dish on a water-levelled ground were performed.

In order to achieve the necessary heat denaturation of the cast formulations, the filled petri dishes were placed at 105 °C for 40 min in the compartment dryer, Kelvitron T6120 (Heraeus Thermo Electron Corporation, Langenselbold, Germany), followed by a post-drying at 23 °C and 50% relative humidity (r.h.), until the equilibrium moisture content was reached.

To determine the real temperature of the native formulations during the first process step and, thus, to monitor the protein denaturation, iButton sensors (Dallas Semiconductor Inc., Dallas, Texas, TX, USA) were positioned in the center of selected petri dishes, recording the temperature-time profile (TTP). The temperature sensor was fully immersed in the formulations. The determined TTP was at least of 80 °C for 2000 s for all samples at the process conditions mentioned above. This TTP ensures, according to Kessler [28], that an irreversible heat induced degree of protein denaturation/unfolding of at least 90% was reached. In addition to that, the on-set denaturation temperature of 70 °C for the major WPI components was exceeded [29].

The main advantages of cast films as produced in this study are the independency of any substrate for characterization and evaluation of the results. Therefore, the dried films were removed from the petri dishes by cutting the edges, followed by carful peeling, until the films were removed without any loss or damage. However, it is not possible to directly compare the results to materials applied by commonly used coating techniques, since no polymer orientation and a significantly slower curing takes place. Nevertheless, it is applicable to compare the results within the same series of trials, as performed in this study.

### 2.3. Equilibrium Moisture Content

To measure the equilibrium moisture content (EMC) the scale, MA30 (Sartorius AG, Göttingen, Germany), was used. WPI-based films were stored at 23 °C and 50% r.h., until they reached a constant weight. The IR-sample dish made of aluminum is tarred, and 0.5 g of the WPI film is placed into it and placed flat. The sample is dried at a temperature of 105 °C, until the mass is constant, for at least 5 min. The water content is given in % (w/w). For every sample, a four-fold determination was performed. Average mean values and standard deviations were calculated and plotted as error bars.

### 2.4. Film Thickness

The film thicknesses of WPI-based films were measured after they reached their respective equilibrium moister content at 23 °C and 50% r.h. by the Precision Thickness Gauge FT3 (Rhopoint Instruments, Bexhill on Sea, UK) at five different positions. The measurement conditions were 23 °C and 50% r.h. The arithmetic average of the film thicknesses are used to determine oxygen permeability (OP), water vapor transmission rate (WVTR) and mechanical film properties, whereas the standard deviations were taken into account for the error propagation, accordingly.

### 2.5. Water Vapor Transmission Rate Measurement

The gravimetric screw cup method, according to the standard DIN 53 122-1, was used to measure the WVTR (water vapor transmission rate) *Q* of the cast films at 23 °C and 50% → 0% r.h.

The WVTR is calculated by the following equation:

(1) $$\text{WVTR}=Q=\frac{24}{t}\times \frac{\text{∆}m}{\text{A}}\times 10^{4}$$

where *t* is the period of time between two weight measurements in h, Δ*m* represents the weight difference between two weight measurements in g and A is the test area in cm^2^. The WVTR values, *Q*, are stated in the unit, g·m^−2^·d^−1^, and converted to the thickness d of 100 µm (*Q*_100_) using the following equation in order to allow direct comparison of different materials independently of the coating thickness:

(2) $$Q_{100}=Q\cdot \frac{d}{100}$$

A four-fold determination was performed in all cases. [30]

### 2.6. Oxygen Permeability Measurement

The OP (oxygen permeability) measurement was performed according to the standard DIN 53380-3 at 23 °C and 50% r.h. by the instrument, Mocon Twin (Mocon Inc., Minneapolis, Minnesota, MN, USA). The WPI films are masked using aluminum films in order to stabilize the samples. In the case of a deviation >10% between two OP values, a third determination was performed. The OP values, *Q*, are given in the unit, cm^3^ (STP)·m^−2^·d^−1^·bar^−1^, and were converted to the thickness, *d*, of 100 µm (*Q*_100_), according to Equation 2, for direct comparison of different materials independently of the film thickness [31].

### 2.7. Tensile Property Measurement

The tensile strength (TS) of WPI-based films was measured by the universal compression-tension testing machine, RM 50 (Doli GmbH Industrie Electronic, Munich, Germany). According to the standard ISO 527, forces were applied to the samples from both sides, until a change in shape or a fracture took place [32].

The cast films were cut into strips of 15 mm width and a length of 70 mm. In order to measure the tensile properties as a function of the thickness, the thicknesses of the films were measured. For each specimen, a five-fold determination was performed, and the arithmetic average and standard deviation were calculated.

The ends of the specimen were clamped by utilizing pneumatic grips in the loading frame. The initial gauge length of the specimen was set to 50 mm. After the traverse stroke and the force were tarred, the specimen was subjected by an applied force using a load cell of 50 N. The specimen was stretched using a testing speed of 100 mm/min. A ten-fold determination was performed for each sample at testing conditions of 23 °C and 50% r.h.

### 2.8. Young’s Modulus Measurement

The Young’s Modulus or the elastic modulus (EM) of WPI films were measured by the universal compression-tension testing machine, RM 50 (Doli GmbH Industrie Electronic, Munich, Germany), in a separate measurement next to the tensile test. According to ISO 527, a sample is subjected to applied forces until an elastic deformation takes place [32]. The Young’s Modulus is the proportional ratio of stress to strain in the elastic region [33,34]. Stripes of 15 mm width and 70 mm length were used. The thickness of every specimen is measured at five different positions, and the arithmetic average and standard deviation are determined in order to measure the elastic behavior of WPI films as a function of the thickness. The specimens were subjected to applied forces using a load cell of 50 N. An initial gauge length of 50 mm was set. The specimens were stretched using a testing speed of 0.5 mm/min at a constant testing condition of 23 °C and 50% r.h. Ten-fold determination was performed.

### 2.9. Surface Energy Measurement

The surface energy of WPI-based films was measured by the contact angle measuring system, G2 (Krüss GmbH, Stephanskirchen/Rosenheim, Germany), using the sessile drop method. This method applies a liquid drop on the surface of a solid to determine the contact angle between the baseline of the drop and the tangent at the drop boundary.

After the glass syringes were refilled by the following testing liquids, double distilled water, diiodo-methane, ethylene-glycol and dimethyl phthalate, the samples were cut into pieces (7.5 mm × 7.5 mm). The samples were positioned on the moveable suction plate, which is connected with a vacuum pump and fixed using tape. A testing drop of 3 µL is induced on the surface of the solid using a dosing rate of 20 µm/min in order to set an ideal screen of the liquid drop at the monitor by help of contrasts, lightning, sharpness, screen size and an external mirror image of the liquid drop. After an ideal screen drop was set, the screen of the inducing liquid drop on the solid surface was frozen after exactly 20 s to measure the contact angle between the baseline, which is adapted to the liquid drop position and the tangent at the drop boundary. The contact angle of each testing liquid was measured at five different positions on the solid surface of five replicates. The arithmetic average and the standard deviation were calculated and shown as error bars. According to Young’s equation, the surface energy can be calculated as follows:

(3) $${\text{σ}}_{s}={\text{γ}}_{\mathrm{sl}}+{\text{σ}}_{l}\cdot \text{cos}\theta$$

where σ_s_ is the surface tension of the solid in mN/m, σ_l_ stands for the surface tension of the liquid in mN/m, γ_sl_ is the interfacial tension between the solid and the liquid in mN/m and cosθ is the contact angle between the surface tension of the liquid and the interfacial tension between the liquid and solid in angular degree. According to the Owens, Wendt, Rabel und Kaelble method, the surface energy is divided into disperse and polar fractions, which were calculated accordingly. Since the cast films are still partly soluble, the measurement had to be performed after a given time and not at equilibrium. Thus, it is not possible to classify the calculated values as absolute surface energy values. However, for comparison of the samples among themselves, it is reasonable to follow this approach in order to characterize the samples and to draw conclusions.

### 2.10. Color Measurement

The color measurement of WPI films was performed by the spectrophotometer, CM-700d (Konica Minolta Sensing, Tokyo, Japan). First of all, the spectrophotometer was calibrated using a white and black calibration device. Using an illuminant D65 and a wavelength range from 400 to 700 nm, a white ceramic plate was measured as the reference. The color was measured at three replicates (films). Average b values and standard deviations were calculated and plotted as error bars. The SCE (Specular Component Excluded) modus was adjusted.

### 2.11. Statistical Analysis

A completely randomized experimental design was used. Thus, the run sequence of the experimental units was determined randomly. The randomization was performed by the computer program, Visual-XSel 12.0 Multivar (CRGRAPH, Munich, Germany). Statistical analyses were performed with the Kolmogorov-Smirnov-Test procedure in Visual-XSel 12.0 Multivar, and the level of significance was defined at 5% for experiments having at least a four-fold determination per sample. This test was applied in order to investigate if a normal distribution adequately describes the sets of data that were achieved. In all cases, the hypothesis of normality was validated. The mean values for OP measurements were calculated from two-fold determination or three-fold determination when the values differed more than 10%. Those data were not tested on a normal distribution, but the minimum and maximum values were given accordingly.

Extreme values of a sample were removed from the data series by the outlier test of Grubbs and the test of David, Hartley and Pearson. The requirement was that the other values were from the same population. This is checked by the test for normal distribution, as mentioned above [35,36]. The multiple *t*-test in Visual-XSel 12.0 was performed to determine any significant difference among experimental treatments at *p* ≤ 0.05 for experiments having at least four replicates.

## 3. Results and Discussions

### 3.1. Equilibrium Moisture Content

The equilibrium moisture content (EMC) was measured at films conditioned at 23 °C and 50% r.h. in order to provide additional information for the evaluation and interpretation of the results gained in this study. Water can cause similar impacts on the mechanical and other physical properties of protein films compared to Gly, since water can easily penetrate the folded protein’s surface to interact with polar amino acids [37].

**Figure 1:**
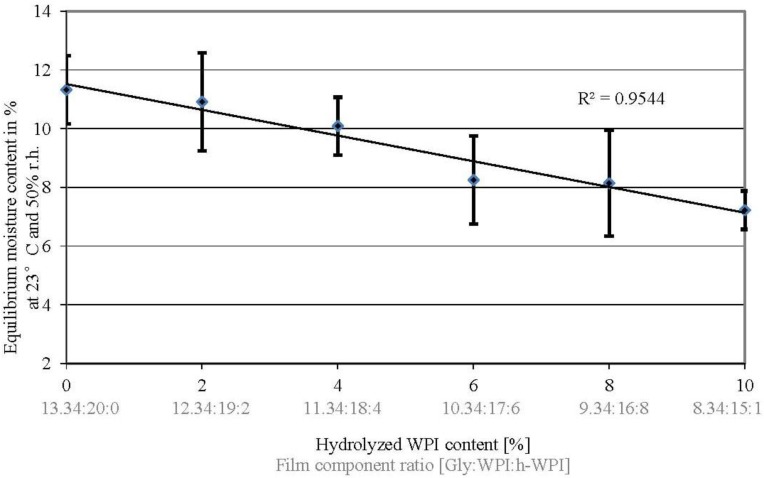
Effect of increased h-WPI content (reduced protein molecular weight (M_W_)) and reduced glycerol contents on the equilibrium moisture content of WPI-based films at 23 °C and 50% r.h.

The results indicate a minor trend towards reduced EMCs when M_W_ and Gly content are decreased (Figure 1). This trend can be explained by the presence of hydroxyl groups in Gly, which facilitate the moisture absorption by their interaction with water molecules through hydrogen bonding [38,39]. However, this trend is not significant (*p* ≥ 0.05) with the exception for the EMC value comparison of 0%, 2% and 4% to 10% (w/w) h-WPI content and the comparison of 0% to 6% (w/w) h-WPI content, where a significant difference was determined (*p* ≤ 0.05). However, the slightly decreased EMCs generally fit to the improved barrier properties of WPI-based cast films, as water can cause similar effects as plasticizers in protein films, such as increasing WTVR and OP values.

### 3.2. Water Vapor Transmission Rate

**Figure 2:**
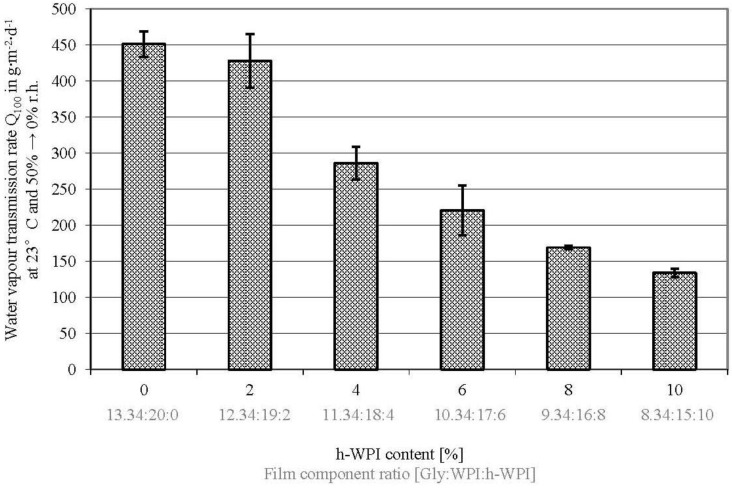
Effect of increased h-WPI content (reduced protein M_W_) and reduced glycerol content on the water vapor transmission rate of WPI-based films.

With exception between 0% and 2% (w/w) h-WPI or 13.34% and 12.34% (w/w) Gly concentration, respectively, a significant decrease in WVTR values of WPI-based films, if M_W_ and Gly content reduction takes place (Figure 2), was observed (*p* ≤ 0.05). These results are consistent with the observations from Sothornvit and Krochta [18] on the effect of Gly concentrations on the WVTR of WPI-based films at constant protein M_W_. In addition to that, it is well known that plasticizers decrease intermolecular forces along polymer chains and increase the polymer free volume, leading to higher permeability values [40]. Even though in this study, the M_W_ and Gly content was decreased at the same time, the WVTR reduction can be traced back to the Gly amount reduction and the lower EMC values. This assumption is confirmed by Schmid *et al.* [25] and several consistent publications [18,41,42] stating that the M_W_ and structure of WPI and its components do not significantly change the WVTR values of WPI-based films. As summarized by Vieira *et al.* [40], this effect of plasticizers on the WVTR is also observed on other biopolymer films, such as polysaccharide and protein- and lipid-based films. However, the overall values determined in this study are not comparable to the values published by Sothornvit and Krochta [18], since another method was used (ASTM E96-92).

The WVTR results show that it is possible to increase the water vapor barrier properties by reducing the Gly content. However, usually, the mechanical properties also dramatically change and lead to brittle films when the plasticizer content is reduced. This was compensated for by the M_W_ reduction, which took place at the same time as the Gly amount reduction. However, the impact on the mechanical properties (TS, EM) will be discussed below.

### 3.3. Oxygen Permeability

**Figure 3:**
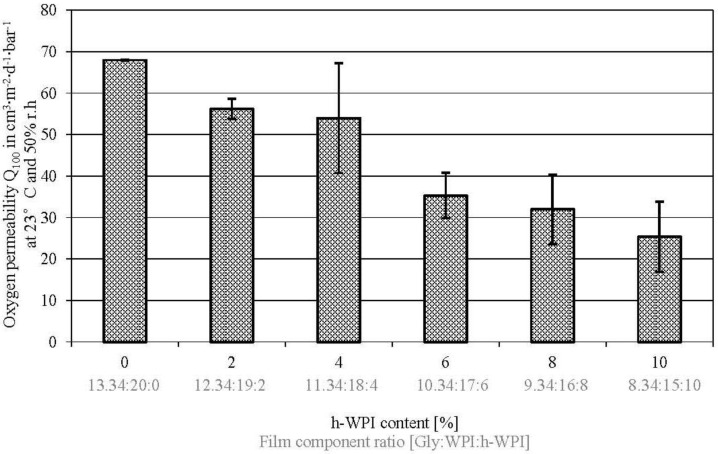
Effect of increased h-WPI content (reduced protein M_W_) and reduced glycerol content on the oxygen permeability of WPI-based films.

There is an observable effect of reducing protein M_W_ and Gly content on the OP values in WPI-based cast films (Figure 3). However, the effect is less distinctive as the effect of reducing the M_W_ and Gly content on WVTR values. The results published by Schmid *et al.* [25] and Sothornovit and Krochta [13], who analyzed the impact of h-WPI content at an equal amount of plasticizer on OP indicate that there are little or no effects of hydrolysed WPI content on OP values. Hence, the observed results in the present study can rather be traced back to the reduced Gly content and the reduced EMC values, rather than on the protein M_W_ reduction. In addition to that, several publications have shown that the WPI composition and structure have no significant effect on film permeability [41,42,43,44]. However, the approach of this work has to be differentiated from those publications, since in this study, hydrolysed and non-hydrolysed WPI were mixed in contrast to the utilization of hydrolysed WPI with different degrees of hydrolysis (DH). Additionally, the Gly content was decreased at the same time as h-WPI content was increased or the protein M_W_ decreased, respectively. Nevertheless, the results of this study confirm that it is possible to take advantage of reducing the Gly content, leading to higher barrier values, while the film flexibility can be maintained, due to the reduction of protein M_W_. The mechanical properties will be discussed below.

### 3.4. Tensile Property

**Figure 4:**
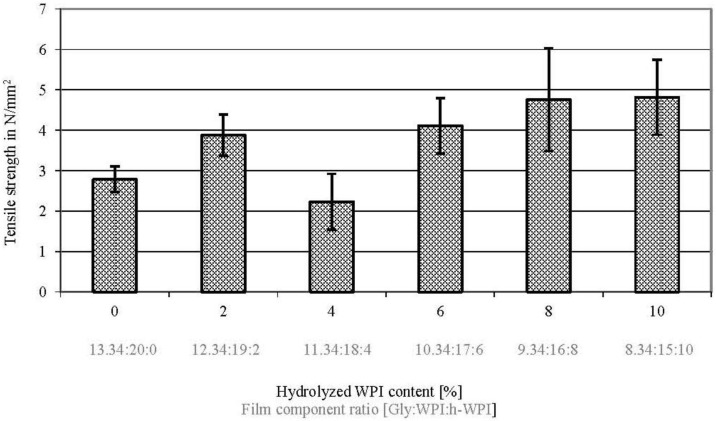
Effect of increased h-WPI content (reduced protein M_W_) and reduced glycerol content on the tensile strength values of WPI-based films.

Tensile strength (TS) properties provide data for the quantification of protein network interactions. In WPI-based films, TS is slightly affected between the comparison of 0% to 2%, 4%, 6%, 8% and 10% and in comparison of 2% to 4% and 10%, as well as in comparison of 4% to 6%, 8% and 10% (w/w) h-WPI content (*p* ≤ 0.05), when the protein M_W_, EMC and Gly content decreases (Figure 4). However, TS is not significantly affected in comparison of 2% to 6% and 8% and comparison of 6% to 8% and 10% (w/w) h-WPI content (*p* ≥ 0.05) when the protein M_W_, EMC and Gly content decreases (Figure 3). These results can be explained by weaker protein network interactions between the shorter chains of hydrolysed WPI in comparison to the longer chains of unhydrolyzed WPI, as shown by Sothornvit and Krochta [17] and confirmed by Schmid *et al.* [25]. Thus, h-WPI is able to act as the internal plasticizer and, thus, compensates the reduction of Gly and EMC as the plasticizer, as shown in this study for several formulations. It was shown that increasing h-WPI content or reducing M_W_, respectively, while reducing Gly at the same time, can lead to similar TS values in WPI-based films, whereas the barrier properties can be increased significantly. Researchers and material developers can benefit from these results in order to improve the barrier properties of WPI-based films and coatings, while maintaining film flexibility.

### 3.5. Elastic Property

**Figure 5:**
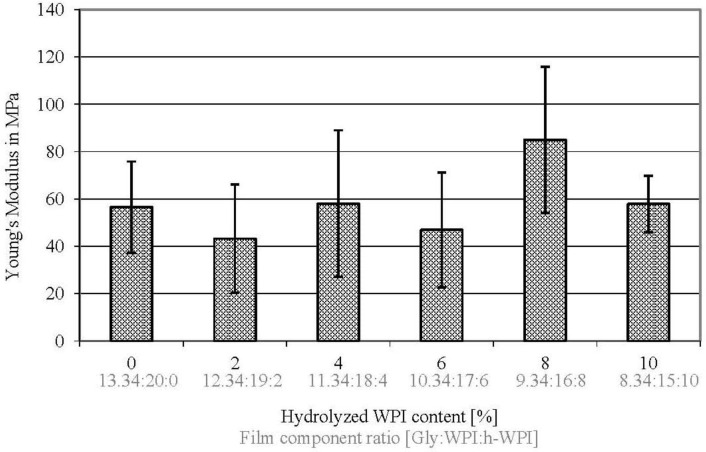
Effect of increased h-WPI content (reduced protein M_W_) and reduced glycerol content on the Young’s Modulus values of WPI-based films.

The higher the Young’s Modulus (EM) value of the material is, the stiffer or, in other words, less flexible a material is [45]. With the exception of the comparison of 8% to 0%, 2%, 6% and 10% (w/w) h-WPI content, the Young’s Modulus (EM) is not significantly influenced (*p* ≥ 0.05) if the M_W_, EMC and the Gly concentration is reduced in WPI-based cast films (Figure 5), leading to maintained film flexibility of the films, while barrier properties are significantly increased. According to the literature, with increasing shorter chains of amino acids by increasing the h-WPI content, the flexibility increases [17,43]. This is because hydrolysed WPI forms weaker intermolecular forces, and thus, EM decreases by higher hydrolysed WPI ratios, leading to more flexible films [25]. In this study, the reduction of Gly followed by lower EMC values was compensated for by the increasing h-WPI content for maintained film flexibility.

### 3.6. Surface Energy

The knowledge of surface energies and, in particular, the polar and disperse part of the surface energy of solids that are going to be coated or printed is an important factor in industrial film coating applications. According to the Owens, Wendt, Rabel and Kaelble method, the surface energy is divided into disperse and polar fractions, whereas the summation of disperse and polar fractions leads to the total surface energy.

**Figure 6:**
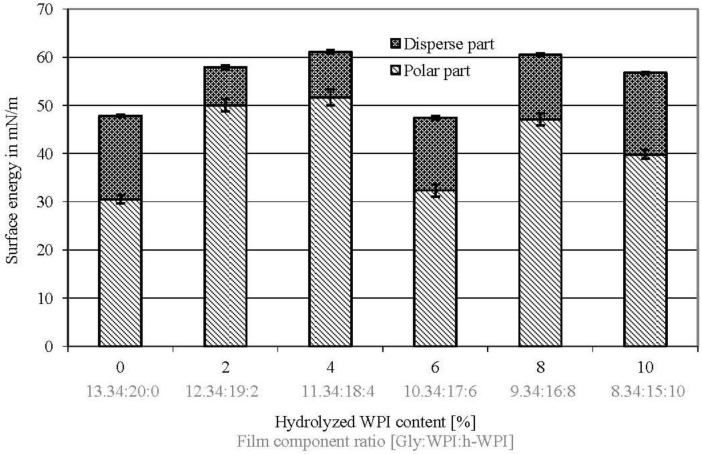
Effect of increased h-WPI content (reduced protein M_W_) and reduced glycerol content on the disperse part, polar part and total surface energy of WPI-based films.

There was neither a significant observable effect of reducing M_W_, EMC and Gly content on the total surface energy values, nor on the disperse and polar part, in WPI-based cast films (Figure 6). Schmid *et al.* [25] showed that the disperse part of the surface energy decreases significantly, and the polar part is significantly increased if the M_W_ is reduced at constant Gly content. They stated that the total surface energy decreased slightly when the h-WPI content was increased from 0% to 1% and from 1% to 2% (w/w). At h-WPI concentrations above 3% (w/w), the surface energy increased. Based on these facts, the results regarding the maintained surface energy values could also be explained by a compensational effect. Increasing the h-WPI concentration (reducing protein M_W_), which increases the polar part by increasing the number of polar end groups in h-WPI in comparison to WPI, is compensated for by the reduced Gly concentration. Gly is known as a hydrophilic plasticizer with 55.4% hydrophilic groups and nine hydrogen bonds [14], thus decreasing the polar part of the surface energy when its concentration is reduced. Consequently, the increasing amount of polar end groups coming from h-WPI is compensated for by the decreasing amount of polar groups from Gly in the WPI-based formulations.

### 3.7. Yellow Coloration

**Figure 7:**
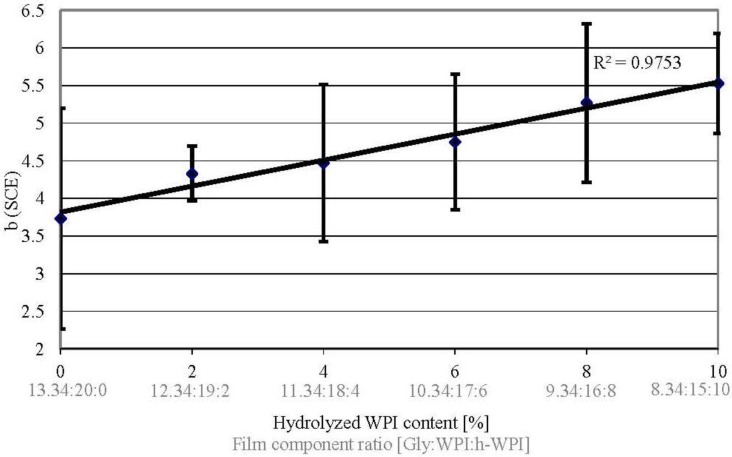
Effect of increased h-WPI content (reduced protein M_W_) and reduced glycerol content on yellow coloration (b-value) of WPI-based films.

Packaging must provide attractiveness to the consumer; therefore, the yellow coloration as an optical property expressed as the b-value of WPI-based films was analyzed. The results show that the b-values increase if protein M_W_ and Gly content decrease (Figure 7). These results are consistent with the results from Schmid *et al.* [25], where the b-value increased from 3.7 to 6.0, when the h-WPI concentration was increased from 0% to 5% (w/w). This marginally, but not significant, increasing yellow coloration might be related to higher amounts of yellowish whey metabolites in the h-WPI used (BioZate 3), which are either not present in the WPI (BiPro) or present in smaller concentrations.

## 4. Conclusions

To the author’s knowledge, this study proves for the first time the possibility of reducing the technically necessary plasticizer concentration by increasing the h-WPI concentration (M_W_ reduction) in formulations where the Gly concentration is reduced at the same time. This led to significantly higher barrier values (*p* ≤ 0.05), while mechanical properties (TS and EM) were maintained (*p* ≥ 0.05). This was possible, since the formulations prepared in this study benefited from increasing flexibility when protein M_W_ is reduced and decreasing free volume in the protein matrixes when Gly concentration is reduced, followed by lower EMC values, leading to decreased OP and WVTR.

However, cost-performance ratios have to be taken into account. This will make the industrial implementation of this approach difficult, since the relatively cheap component, Gly, is partially substituted by h-WPI, which is at least ten-times more costly than Gly. The same applies to protein M_W_ reduction, since WPI is generally cheaper than h-WPI.
